# Self-Care Instruments to Measure Nutrition Practices in Children and Parents: Psychometric Analysis

**DOI:** 10.3390/nu13072242

**Published:** 2021-06-29

**Authors:** Lisa R. Pawloski, Jean B. Moore, Patricia Treffinger, Heibatollah Baghi, Kathleen Gaffney, Sonia Jaimovich, Cecilia Campos, Kevin M. Curtin

**Affiliations:** 1 Department of Geography, College of Arts and Sciences, The University of Alabama, Tuscaloosa, AL 35487, USA; kmcurtin@ua.edu; 2 College of Health and Human Services, George Mason University, Fairfax, VA 22030, USA; jmoore@gmu.edu (J.B.M.); hbaghi@gmu.edu (H.B.); Kgaffney@gmu.edu (K.G.); 3 School of Nursing, ECPI University Roanoke, Roanoke, VA 24012, USA; RoPTreffinger@ecpi.edu; 4 School of Nursing, Pontifica Universidad Catolica de Chile, Macul 7810000, Chile; sjaimovi@uc.cl (S.J.); ccampos@uc.cl (C.C.)

**Keywords:** Orem’s self-care, nutrition behavior instrument, children, parents, Chile

## Abstract

The purposes of this study were to evaluate the psychometric properties of English and Spanish instruments that measure the nutrition behavior and practices of children and their parents. Orem’s self-care deficit nursing theory was used in this methodological study. A convenience sample of 333 children and 262 mothers participated from two schools in Washington, D.C. and two schools in Santiago, Chile. Principal component analysis indicated three component per instrument corresponding to Orem’s Theory of operations demonstrating construct validity of the instrument. The study findings showed evidence for validity and reliability of the English and Spanish versions and indicated that the instruments appropriately represented Orem’s operations. The results have implications for the development of health behavior measurement instruments that are valid, reliable, designed for children, culturally appropriate, and efficient. Measuring the nutrition behavior of children and parents is critical for determining the effectiveness of nutrition intervention programs. Furthermore, instruments are needed so that researchers can compare corresponding child and parent behaviors or compare behaviors across cultures.

## 1. Introduction

Measuring the nutrition behavior of children and parents is critical for determining the effectiveness of nutrition intervention programs. The incidence of obesity in children has doubled since the 1960s [1] and under-nutrition is still a major concern [2]; therefore, developing and evaluating intervention programs is essential. In order to determine effective programs, measurement instruments need to be developed that are valid, reliable, designed for children, culturally appropriate, and efficient [3]. Furthermore, instruments are needed so that researchers can compare corresponding child and parent behaviors or compare behaviors across cultures.

In this study, the researchers targeted the measurement of child and parent nutrition behavior. To this end, the researchers have reviewed existing instruments that measure the nutrition-related behaviors of children and their parents, revised two child and parent instruments, and described how the instruments have been translated into Spanish. Finally, the instruments were tested in U.S. and Chilean samples, and the psychometric properties of the English and Spanish versions of the instruments were compared. Psychometric properties refer to the validity (the extent to which the measure suits its purpose) and reliability (the extent to which the measure is reproducible) of the measurement tool.

### 1.1. Background and Conceptual Framework

#### 1.1.1. Conceptual Framework

Orem’s self-care deficit nursing theory [4] was the theoretical framework for this study. According to Orem’s theory, individuals perform practices that promote and maintain their health state (self-care) or the health state of others such as children (dependent-care). Orem divided self-care practices into three categories of operations, some of which are cognitive and others of which are psychomotor. These operations are incorporated into the nutrition instruments presented here. Each instrument item was developed to measure one of the categories of Orem’s self-care Estimative (acquiring knowledge and obtaining information), Transitional (planning and making decisions), and Productive (taking action and evaluating) operations [4,5].

#### 1.1.2. Literature Review

In a recent literature review, it was found that few studies have tested instruments that measure nutrition behaviors in children. Databases searched included Cumulative Index to Nursing and Allied Health Literature CINAHL (a database for Nursing research journals), Medline, Cochrane Systematic Reviews, and Digital Dissertations for 1999–2019 using the keywords of children, nutrition, and measurement. Of the 17 studies that performed psychometric testing on health behavior instruments, only five were found to have a nutritional component and were used with healthy children. Two of the five studies that involved children assessed instruments originally developed for use with adults [6,7]. One study evaluated instruments focused on parental feeding practices [8], and another was specific to children with cachexia and malnutrition [9]. The three other studies evaluated instruments that were originally developed for use with children [10,11,12].

Hendricks et al. [7] performed psychometric testing on the Adolescent Lifestyle Profile (ALP), which was based on the Health Promoting Lifestyle Profile II (HPLP II) originally created for adults. The HPLP II adult version was revised following a pilot test with adolescents. The revised instrument was then tested on 207 children from a convenience sample at five middle schools. The final version of the ALP had 42-Likert-type scale questions and six subscales, with only nine questions related to nutrition. The nutrition subscale reliability coefficient alpha was found to be 0.67 (*n* = 206). Psychomotor and cognitive behaviors were addressed; however, there is no matching instrument to measure parent behaviors for participating children, there were no results presented comparing before and after behaviors, and only the original adult version had been translated to Spanish.

Similarly, the Fumagalli et al. [6] study was conducted to validate the Food Frequency Questionnaire (FFQ) for use in children, although it was originally developed to measure nutritional intake in adults. A total of 188 children aged 5 to 10 years were randomly selected from public schools in Brazil. In order to determine the reliability of the FFQ, parents of the children completed the FFQ questionnaire, and then were asked to record in a food diary all foods and beverages their children consumed over a three-day period. Nutritional values of the dietary intake reports and the FFQ were calculated and analyzed by the researchers to determine the correlation between the nutrient intake measurements from the FFQ and the nutrient intake measurements from the three-day diet records. Results indicated that most nutrient measurements that were derived from the FFQ were statistically higher than those derived from dietary records, and it was determined that energy was overestimated by 336.5 kcal by the FFQ. The researchers concluded that an adjustment to portion sizes on the FFQ may be necessary for use with children in future studies. The instrument included input from mothers of participants and has been translated into Portuguese. There was no discussion of results that compared before and after behaviors, and no cognitive behaviors were discussed.

Three studies in the literature were related to instruments originally developed for use in children [10,11,12]. In the Anderson et al. [10] study, a questionnaire designed to assess the knowledge of applied nutrition in children who participated in an after-school cooking club was tested in England on 98 children, aged 11 years old. The instrument consisted of 36 questions in three domains, of which half were in the Knowledge of Applied Nutrition (KN) domain. The remaining 18 questions regarded the Knowledge of Food Preparation (KP) domain and the Perceived Confidence in Cooking Skills (PC) domain. Only the PC domain of the instrument involved behaviors, because it required children to evaluate their cooking skills. The researchers concluded that the instrument met the criteria for reliability and validity and was suitable for measuring changes associated with nutrition interventions for improving dietary knowledge. There was no matching parent version of the instrument, no Spanish version was discussed, and no findings for before and after behavioral comparisons were noted.

Similar to the Fumagalli et al. [6] study, the Thiagarajah et al. [12] investigation compared a questionnaire to measure children’s nutrition knowledge and behaviors with the dietary recall reports from participants as a method to determine instrument validity. A total of 121 fourth-grade students in Indiana completed the School Physical Activity and Nutrition (SPAN) questionnaire and recorded a 24 h dietary recall (used as the criterion measure. It was also found that some of the food items showed a gender difference in validity. The SPAN instrument did not include a parent version, a Spanish language version, or results for before and after comparisons.

The instruments most similar to the work presented here were designed to measure self-care behaviors, specifically, nutrition self-care practices in English- and Spanish-speaking children and dependent-care practices of their parents [11]. The Adolescent Nutrition Self-Care Questionnaire (ANSCQ) and the Parent Nutrition Dependent-Care Questionnaire (PNDCQ) were developed and tested originally in English and then translated into Spanish using the methodology suggested by Carlson [13]. The English version was pilot-tested on 32 girls, aged 10–18 years old, and 29 mothers. The Spanish version was tested in Nicaragua on 88 girls and 29 mothers. Both versions of the ANSCQ instrument consisted of 37 Likert-type scale items based on Orem’s operations. The PNDCQ instrument also consisted of 37 Likert-type scale items intended to measure nutrition dependent-care operations that parents perform for children. Each item on the parent questionnaire corresponded to the same item on the child questionnaire. Results of psychometric analysis indicated that the coefficient alpha for both versions of both instruments was above the recommended threshold of 0.70 [14], except for the English version of the parents’ post-test, where the removal of one item resulted in a coefficient of 0.72. The researchers noted that more items should be added to the instruments to reflect the operations regarding acquiring knowledge, making decisions, and planning actions (reflecting both Estimative Operations and Transitional Operations). In comparison to the reported studies found in the literature review, the ANSCQ and the PNDCQ measure several previously unaddressed nutrition targets, such as matching self-care practices of parents and children; English and Spanish versions; a comparison of pre- and post-test results; and the inclusion of both cognitive and psychomotor behaviors.

In conclusion, there are few instruments measuring nutrition behaviors of children: only two have been translated into Spanish, and only one included a corresponding parent version and the identification of self-care practices.

### 1.2. Purpose

Impact Statement: Measuring the nutrition behavior of children and parents is critical for determining the effectiveness of nutrition intervention programs. In order to determine effective programs, measurement instruments need to be developed that are valid, reliable, designed for children, culturally appropriate, and efficient. Furthermore, instruments are needed so that researchers can compare corresponding child and parent behaviors or compare behaviors across cultures. This study provides evidence for validity and reliability for the MIN-C and the MIN-P nutrition behavior instrument. Furthermore, principal component analysis provides support for the instruments’ measurement of Orem’s self-care operations.

The purpose of this study was to evaluate the psychometric properties of English and Spanish versions of two previously developed instruments that measure the nutrition self-care practices of children and the nutrition dependent-care practices of their parents. A further purpose was to compare the psychometric properties of the English and the Spanish versions of these instruments. We termed the instruments the Index of Nutrition—Child (IN-C) and the Index of Nutrition—Parent (IN-P).

### 1.3. Research Questions

1. What are the psychometric properties of the Index of Nutrition—Child (IN-C), English and Spanish versions?
2. What are the psychometric properties of the Index of Nutrition—Parent (IN-P), English and Spanish versions?

## 2. Materials and Methods

### 2.1. Design

The researchers used a methodological design to revise the two instruments (IN-C and IN-P) to better measure children and parents’ nutrition self-care and dependent-care practices, respectively. Specifically, although the previous instruments measured Productive Operations, there were fewer items that measured Estimative and Transitional Operations. To improve the content validity of the instruments, thirteen items were written and added to the original 37-item instruments. Each instrument was translated into Spanish and back-translated into English by bilingual native Chilean experts who were familiar with the cultural nuances of the instrument language. Psychometric properties of the instruments were examined, including validity evidence (i.e., content validity, construct validity, examination of bias/cultural appropriateness, and translation verification) and internal consistency reliability evidence (using Cronbach’s alpha coefficients). Content validity is discussed in the instruments section, translation and bias/cultural appropriateness in the procedure section, and reliability and construct validity evidence in the results section.

### 2.2. Sample

A convenience sample of four groups of respondents participated in this study. A total of 126 children from two schools in Washington D.C. and their 105 mothers completed the English version of the child and parent instrument, respectively. In addition, 207 children from two schools in Santiago, Chile, and their 157 mothers completed the Spanish version of the child and parent instrument, respectively. Characteristics of the sample are summarized in Table 1. Participants all signed informed consent and assent agreements. In both settings, children and parents were recruited from schools using informational flyers and informational meetings. Children were given pens as incentives and both parents and children were provided with information about the child’s nutrition status. Additional details of the recruitment of participants from the Washington, D.C. sample are also noted in Moore et al. [15].

### 2.3. Instruments

The instruments developed here used the instrument developed in Moore et al. [11] as a foundation, with 13 additional items specifically designed to better measure the three domains of self-care operations. These new instruments were termed the Moore Index of Nutrition—Child, and the Moore Index of Nutrition—Parent.

#### 2.3.1. The Moore Index of Nutrition—Child (MIN-C)

This instrument has 50 items in a five choice, Likert-type scale format. The first 42 items measure the frequency of behaviors ranging from 1 (never) to 5 (always). The last 8 items, which ask about the frequency of intake of specific foods, employ a different scale from 1 (not at all) to 5 (3 or more times a day). Higher scores indicate more frequent self-care practices.

#### 2.3.2. The Moore Index of Nutrition—Parent (MIN-P)

This instrument has 50 items in a five choice, Likert-type scale format with items formatted as described for the child questionnaire. Higher scores indicate more frequent dependent-care practices. Each parent questionnaire item is matched with a corresponding item on the child questionnaire.

Evidence for content validity involved having researchers with expertise in Orem’s theory write items that reflected Orem’s operations and the United States Department of Agriculture dietary guidelines [16]. Previous and new items were evaluated by six experts in nursing, nutrition, or psychometrics.

### 2.4. Procedure

The study was approved by two university human subjects review committees, (Blinded University A and University B), a charter school committee, and two school committees in Chile. The researchers visited two Washington, D.C. schools and two Santiago schools, and met with the administrators, teachers, and children in each school to explain the study. Permission forms and questionnaires for parents were sent home with the children and assent forms were given to the children. Once the researchers received both a signed assent form and corresponding parent consent form, the child and parent were included in the study and completed the questionnaires.

### 2.5. Translation Procedure

The researchers followed the guidelines for instrument translation suggested by Carlson [13]. The instruments were translated into Chilean Spanish by a bilingual native Chilean nursing faculty member. Subsequently, the Spanish items were translated back into English by a bilingual Chilean in collaboration with an English-speaking researcher who verified the accuracy of the translation. Each Spanish translator was familiar with Orem’s theory, the constructs of self-care operations, and the culture in which the instruments would be used. Each translator was aware of the intended future use of the instruments and had previous experience with research. To further ensure content validity, the researchers tested the instruments using methodology suggested by Evers [17] and Nunnally and Bernstein [14] using twelve suggested stages including pilot testing, content validity, item analysis, and reliability. After each stage, the researchers revised the instrument. Additional details on these procedures can be found in Moore et al. [11].

### 2.6. Data Analysis

We performed principal component and confirmatory factor analysis (CFA) using a varimax rotation on the items. The analyses were performed using SAS, Statistical Analysis Systems, v9.4 software, Cary, NC, USA. The weighted least squares technique was used as the estimation method for the CFA. Various indices were used to assess the fit of the model, including goodness-of-fit index (GFI), adjusted goodness-of-fit index (AGFI), comparative fit index (CFI), and normed fit index (NFI). The internal consistency reliability of scores for the questionnaire items were estimated using Cronbach’s alpha. The present study used Cronbach’s alpha because the method assesses errors due to sampling of the content domain. In other words, the internal consistency reliability is the degree to which the items are representative of the domain that is being measured. Although the minimum recommended standard for Cronbach’s alpha varies among psychometricians, Nunnally and Bernstein [14] recommend an acceptable value of 0.70.

## 3. Results

In this section, the researchers report evidence for reliability and construct validity. The researchers conducted principal component analysis for the first 42 of the 50 items, because the last 8 items on these instruments used a different scale of measurement. The presentation for construct validity includes: (a) the number of reliable and interpretable components present among the 42 items listed on the instruments in both the English and the Spanish versions, (b) the total variance accounted for by the components, (c) the interpretation of these components, and (d) the means, standard deviations, and alpha coefficients for each component in each instrument.

Validity evidence appropriate for the present study was drawn from the internal structure of the MIN-C and MIN-P for both the English and Spanish versions. The analyses are intended to demonstrate the degree to which the relationships among items in the instruments measure the construct on which the score interpretation is based. The theoretical framework for a measuring instrument may include one dimension of the proposed construct or several homogeneous dimensions.

Confirmatory factor analysis (CFA), in addition to principal component and test of fit, provides very strong evidence for construct validity. Construct validity of the questionnaires was investigated using a principal component analysis with varimax rotation [18] and Kaiser normalization. The argument has been made that with varimax rotation, each component loading value is high on a small number of variables and low on the other variables, making the results easier to interpret. It is expected that items in the questionnaires would load on the following three components or dimensions: Estimative, Transitional, and Productive self-care operations that represent self-care practices in Orem’s self-care deficit nursing theory (see Table 2 for a summary of the component loadings for each instrument).

### 3.1. Principal Component Analysis for the MIN-C, English Version

In the principal component analysis for the MIN-C, English Version, the scree plot leveled off at three or four components, accounting for 42.91% of the variance and 45.61% of the variance, respectively. The three-component solution was the most interpretable (Table 2). With this solution, Component 1 accounted for 28.04% of the variance, Component 2 accounted for 8.67% of the variance, and Component 3 accounted for 4.76% of the variance.

With the three-component solution, Component 1 was composed primarily of gathering information and some choosing (representing Estimative Operations), Component 2 was composed of taking action behaviors related to healthy behaviors (representing Productive Operations), and Component 3 was composed of decision making and taking action related to unhealthy behaviors (mainly representing Transitional operations).

### 3.2. Principal Component Analysis for the MIN-C, Spanish Version

In the principal component analysis for the MIN-C, Spanish Version, the scree plot leveled off at three or four components, accounting for 33.32% of the variance and 37.17% of the variance, respectively (somewhat lower than for the English version). The three-component solution was the most interpretable (Table 2). With this solution, Component 1 accounted for 21.58% of the variance (somewhat lower than for the English version), Component 2 accounted for 6.18% of the variance (somewhat lower than for the English version), and Component 3 accounted for 5.55% of the variance (slightly higher than for the English version).

With the three-component solution, Component 1 was composed primarily of gathering information and some choosing (primarily representing Estimative Operations, as in the English version), Component 2 was composed of taking action with an emphasis on healthy behaviors (representing Productive Operations, as in the English version), and Component 3 was composed of taking action and choosing between healthy and unhealthy behaviors (representing both Productive and Transitional Operations, similar to the English version).

### 3.3. Principal Component Analysis for the MIN-P, English Version

In the principal component analysis for the MIN-P, English Version, the scree plot leveled off at either three or four components with three components accounting for 59.25% of the variance and four components accounting for 62.45% of the variance. The three-component solution (Table 2) was the most interpretable. With this solution, Component 1 accounted for 48.36% of the variance, Component 2 accounted for 6.28% of the variance, and Component 3 accounted for 4.6% of the variance.

With the three-component solution, Component 1 was composed primarily of taking action with some choosing (representing Productive and Transitional Operations), Component 2 was composed of choosing with some taking action (primarily representing Transitional Operations), and Component 3 was composed of seeking information behaviors (representing Estimative Operations).

### 3.4. Principal Component Analysis for the MIN-P, Spanish Version©

In the principal component analysis for the MIN-P, Spanish Version, the scree plot leveled off at either three, four, or five components, with three components accounting for 37.20% (vs. 59.25% for the English version) of the variance, four components accounting for 41.63% (vs. 62.45% for the English version) of the variance, or 45.69% of the variance. The three-component solution was the most interpretable (Table 2). With this solution, Component 1 accounted for 20.90 % of the variance (vs. 48.36% for the English version), component 2 accounted for 9.75% of the variance (vs. 6.28%), and Component 3 accounted for 6.55% (vs. 4.6%) of the variance.

With the three-component solution, Component 1 was composed primarily of gathering information (representing Estimative Operations) compared to the English version (representing Productive and Transitional Operations). Component 2 was composed of taking action and some planning regarding healthy behaviors similar to the English version. Component 3 was composed of taking action and choosing related to unhealthy behaviors (Productive and Transitional) vs. Estimative Operations in the English version. The Kaiser–Meyer–Olkin (KMO) measure of sampling adequacy is a statistic that indicates the proportion of variance in the variables that might be caused by underlying factors. The reported values of KMO for the principal component analysis (for all four instruments) in the present study were greater than 0.70, which indicates that a principal component analysis was appropriate with the data.

Using confirmatory factor analysis (CFA), we evaluated the goodness of fit of the models using several fit indices. The values of GFI, AGFI, CFI, NFI (for four instruments) were in the range of 0.89–0.96, 0.88–0.91,0.90–0.94 and 0.88–0.90, respectively, suggesting a good fit of the models. Overall, these results show that the proposed models were reasonably fitted to the data.

### 3.5. Reliability Evidence

Estimates of the internal consistency reliability for the total questionnaire and for each component were determined using Cronbach’s alpha coefficient [14]. The internal consistency reliability is the degree to which the items are representative of the components being measured [19]. Although the minimum standard for Cronbach’s coefficient alpha varies among psychometricians, Nunnally and Bernstein [14] recommend a stringent standard of 0.70. Cronbach’s alpha coefficients for the English and Spanish questionnaires for children and parents were all above 0.88 and are shown in Table 3.

## 4. Discussion

The goal of this study was to evaluate, test, and compare the results of the English and Spanish versions of the MIN-C and the MIN-P designed to measure children’s and parents’ nutritional self-care and dependent-care practices, respectively. Orem’s self-care deficit nursing theory [4] was useful for identifying cognitive as well as psychomotor behaviors related to nutrition. The researchers constructed the questionnaire items to reflect these behaviors.

### 4.1. Comparison of Instruments

Each instrument in both versions had component solutions that reflected the three operations in Orem’s theory. Comparing the principal component analysis for the English and Spanish versions of the MIN-C, the Spanish version accounted for a somewhat lower percentage of the variance than the English version (33.32% vs. 42.91%, respectively). Similar to the English version, the three-component solution was the most interpretable. As shown in Table 2, in both versions, Component 1 primarily represented Estimative Operations, Component 2 primarily represented Productive Operations, and Component 3 represented Transitional Operations with some Productive Operations added on the Spanish version.

Comparing the principal component analysis for the English and Spanish versions of the MIN-P, the Spanish version accounted for a lower percentage of the variance with the three-component solution (37.20% vs. 59.25%): Estimative operations were represented in Component 1 in the Spanish Version and Component 3 in the English version. Both Productive and Transitional Operations were represented in the other two components, with Component 2 consisting of healthy nutrition behaviors for both versions.

### 4.2. Comparison to Other Literature

When compared to other published instruments that measure nutrition behavior, the MIN-C and the MIN-P are designed to measure self-care practices, compare children and parent practices, compare English-speaking and Spanish-speaking populations, and assess cognitive and psychomotor behaviors.

Although many other instruments have been used to measure nutrition behavior, most are focused on dietary consumption and energy expenditure. The application of self-care practices used in the MIN-C and MIN-P are ideal for the development of nursing and nutrition education interventions. For example, Moore et al. (2009) used the MIN-C instrument to assess the impact of a nutrition intervention program among elementary students in Washington, D.C. The results showed improved scores for self-care practices, although physical assessment data (BMI-for-age) did not result in improved indicators. Healthy lifestyle is a lifelong process; therefore, the use of self-care instruments is key to understanding that change is occurring. Additionally, such assessment can assist in developing and monitoring healthy eating, even at a young age. Self-care can be viewed as a multifaceted and ever-developing process, which can assist not with just developing nutrition interventions but also for framing the rationale and processes for lifelong choices.

In addition to the advantages of the instrument in lifelong behavior change, one major challenge for global health and using the instrument in non-English speaking environments is ensuring that such instruments are culturally relevant beyond the initial formal translation. Spanish is spoken in much of the world; however, the nuance of the language varies significantly among cultures and regions. Thus, the validity of such an instrument in Chile may be different from that in Nicaragua, and understanding the validity evidence for this specific instrument is key for future nutrition studies in these population. Furthermore, this study supports the literature in suggesting that the development of new tools is not necessarily useful, but validating and comparing existing tools within specific populations is more important [20].

Validity evidence for other similar instruments included the study by Hendricks et al. [7], who reported factor analysis results for the Adolescent Lifestyle Profile, and Thiagarajah et al. [12], who tested for correlations of food consumption items (Table 1). Reliability coefficient alpha results for the MIN-C and the MIN-P were significantly stronger than those reported for other child nutritional behavior instruments (Table 4). In addition, only one other study in the literature review included parent participation [6]. Although two studies were found that included Spanish or other language versions of their instrument [6,11], only the Moore et al. [11] study and the present study compared reliability results between the two language versions of their instruments. Additionally, there was a lack of evidence in the literature reviewed regarding instruments that measure change in behavior, with the exception of the Moore et al. [11] study and the present study. Finally, only the Hendricks et al. [7] study, the Moore et al. [11] study, and the present study have reported using a theoretical approach in the development of their instruments.

## 5. Limitations, Recommendations, and Conclusions

Three of the four schools in this study (two in the United States and one in Chile) were in lower-income areas which may limit the applicability of the findings to other populations. Principal component analysis requires substantial sample sizes. Tabachnick and Fidell suggest that the datasets should include at least 300 cases for a principal component analysis to return reliable components. The estimated reliability of components is fair with a sample size of 200 [21]. In this study, sample sizes ranged from 105 to 207, smaller than advisable in most cases. Perhaps as a result of the small sample sizes, the percentage of the variance accounted for was less than ideal.

The researchers recommend the following for future research:

1. Continue to test psychometric properties of these instruments in larger samples;
2. Conduct further examination of the relationship of children’s self-care practices and mothers’ dependent-care practices;
3. Compare self-care and dependent-care practices in other English- and Spanish-speaking populations.

Initial psychometric testing of the English and Spanish versions of these instruments indicates that there is evidence for validity and reliability for the MIN-C and the MIN-P. Principal component analysis and confirmatory factor analysis provided support for the instruments’ measurement of Orem’s self-care operations.

## Figures and Tables

**Table 1:** Characteristics of the study sample.

Characteristics		Washington, D.C. Total *n* (%)*	Santiago, Chile Total *n* (%)*
School	School 1	64 (50.8)	104 (50.2)
	School 2	62 (49.2)	103 (49.8)
Age	8 years		6 (2.9)
	9 years	40 (31.7)	45 (21.7)
	10 years	62 (49.2)	41 (19.8)
	11 years	24 (19.0)	37 (17.9)
	12 years		42 (20.3)
	13 years		20 (9.7)
	14 years		4 (1.9)
	15 years		11 (5.3)
	16 years		1 (0.5)
Gender	Male	46 (36.5)	88 (42.5)
	Female	80 (63.5)	119 (57.5)
Ethnicity	African American	113 (93.4)	
	Hispanic		207 (100)
	Other	8 (6.6)	
Health problems	Yes	35 (29.7)	48 (23.1)
	No	83 (70.3)	105 (50.7)
	Missing		54 (26.1)
Sports participation	Yes	62 (52.5)	104 (50.2)
	No	56 (47.5)	50 (24.2)
	Missing		53 (25.6)
Age of guardian	<30	35 (33.3)	2 (1.3)
	31–40	48 (45.7)	85 (54.1)
	41–50	22 (21.0)	66 (42.0)
	51–60	0 (0)	4 (2.5)
Highest level of education of parent	Did not complete high school	7 (6.5)	16 (10.2)
	High school diploma	37 (34.3)	49 (31.2)
	Some college	31 (28.7)	11 (7.0)
	College degree	26 (24.0)	67 (42.7)
	Graduate school	7 (6.5)	6 (3.8)
	Missing	4 (2.5)	8 (5.1)

Children—Washington, D.C. (*n* = 126), Children—Santiago, Chile (*n* = 207); Mothers—Washington, D.C. (*n* = 105), Mothers—Santiago, Chile (*n* = 157); * Due to rounding, not all percentages equal 100%.

**Table 2:** Principal component analysis.

Item #	Statement		Child English	Child Spanish	Parent English	Parent Spanish
1	I plan my meals so that they are healthy		0.568	0.523	0.735	0.390
2	I read about nutrition and books, magazines and other media		0.743	0.576	0.471	0.742
3	I choose to drink soda instead of water		0.496	0.668	0.651	0.712
4	I study food labels to learn about nutrients in food		0.543	0.515	0.528	0.502
5	I learn about healthy food from watching TV		0.618	0.510	0.601	0.654
6	I suggest healthy food for my family to buy		0.502	0.466	0.652	0.603
7	I eat foods that are good for me even though I do not like them		0.571	0.271	0.690	0.474
8	I try new foods		0.430	0.393	0.658	0.395
9	I ask my teacher about healthy foods I should eat		0.562	0.593	0.337	0.568
10	I eat foods containing iron		0.464	0.578	0.654	0.583
11	I choose to eat foods that contain vitamins		0.592	0.520	0.659	0.680
12	If I think I’m gaining weight I eat fewer sweets		0.416	0.331	0.447	0.333
13	I ask my grandparents about healthy eating		0.502	0.703	0.601	0.531
14	When I buy a snack I choose a soda rather than fruit		0.683	0.690	0.635	0.470
15	I put a lot of salt on the food I eat		0.662	0.467	0.634	0.445
16	I eat the same foods every day		0.611	0.242	0.655	0.290
17	Nurses teach me about healthy foods		0.542	0.308	0.330	0.414
18	I make sure the water I drink is clean		0.427	0.219	0.581	0.553
19	I study nutrition in school		0.338	0.394	0.550	0.410
20	I ask my mother which foods are healthy		0.454	0.471	0.501	0.515
21	I eat foods that are good sources of Vitamin C		0.522	0.621	0.621	0.750
22	I wash fruit before eating it		0.497	0.311	0.586	0.454
23	I make sure that the meat I eat is cooked enough		0.479	0.282	0.612	0.376
24	I talk to my friends about which healthy foods we should eat		0.607	0.593	0.638	0.686
25	I eat foods containing protein in every meal		0.519	0.718	0.598	0.684
26	I try to eat food and drink beverages with calcium		0.524	0.641	0.717	0.581
27	I try to eat food and drink beverages with calcium		0.556	0.619	0.734	0.587
28	I consider whether my meals have enough protein		0.602	0.714	0.649	0.535
29	I eat breakfast every day		0.538	0.202	0.752	0.267
30	I drink soda instead of fruit juices		0.707	0.574	0.784	0.720
31	I would choose to eat sweets instead of a piece of fruit		0.734	0.506	0.724	0.770
32	I think about eating healthy		0.472	0.497	0.686	0.476
33	I drink coffee with meals		0.566	0.264	0.763	0.354
34	I choose to eat foods that are low in fat’s		0.663	0.497	0.706	0.423
35	I obtain information about nutrition from the Internet		0.410	0.624	0.565	0.583
36	I read advertisements about nutritious foods		0.544	0.689	0.567	0.791
37	I eat a variety of foods		0.406	0.632	0.752	0.488
38	I drink eight glasses of liquid every day		0.732	0.233	0.667	0.274
39	I choose to eat chips instead of fruits			0.602	0.782	0.660
40	I read about nutritious food in magazines or newspapers		0.360	0.687	0.656	0.800
41	I help my family select food to buy		0.756	0.411	0.699	0.411
42	I ask other adults questions about healthy eating		0.457	0.721	0.607	0.702
# of iterations for rotation convergence: Child English (6), Child Spanish (5), Parent English (15), Parent Spanish (5)						
**Component Key**						
**Instrument**	**Component I**	**Component II**	**Component III**			
Child English	Estimative	Productive	Transitional			
Child Spanish	Estimative	Productive	Transitional and Productive			
Parent English	Productive and Transitional	Productive and Transitional	Estimative			
Parent Spanish	Estimative	Productive and Transitional	Productive and Transitional			

**Table 3:** Means, standard deviations (SD), and alpha coefficients for the study questionnaires.

	Moore Index of Nutrition—Child					
	English			Spanish		
	Mean	SD	Alpha	Mean	SD	Alpha
Component I	45.29 (17 items)	14.41	0.871	47.74 (19 items)	13.12	0.878
Component II	45.50 (15 items)	15.08	0.895	49.16 (13 items)	7.48	0.749
Component III	34.69 (10 items)	10.71	0.855	35.33 (10 items)	6.64	0.714
	**Moore Index of Nutrition—Parent**					
	**English**			**Spanish**		
	Mean	SD	Alpha	Mean	SD	Alpha
Component I	46.21 (15 items)	19.39	0.961	43.10 (16 items)	11.92	0.884
Component II	64.50 (19 items)	20.39	0.947	62.11 (15 items)	8.40	0.838
Component III	18.97 (8 items)	7.89	0.863	44.44 (11 items)	6.09	0.715

**Table 4:** Alpha coefficients on MIN-C and MIN-P, English and Spanish Versions.

Instrument	Number of Items	Children	Mothers
English Version	42	0.934	0.973
	50	0.941	0.978
Spanish Version	42	0.894	0.894
	50	0.890	0.888

## Data Availability

Not applicable.

## References

[1] World Health Organisation Obesity and Overweight. https://www.who.int/news-room/fact-sheets/detail/obesity-and-overweight.

[2] Amigo H., Bustos P., Leone C., Radrigán M.E. (2001). Growth Deficits in Chilean School Children. J. Nutr..

[3] National Research Council (US) and Institute of Medicine (US) (2004). Children’s Health, The Nation’s Wealth: Assessing and Improving Child Health.

[4] Orem D.E. (2001). Nursing Concepts of Practice.

[5] Dennis C. (1997). Self-Care Deficit Theory of Nursing: Concepts and Applications.

[6] Fumagalli F., Monteiro J.P., Sartorelli D.S., Vieira M.N.C.M., Bianchi M.D.L.P. (2008). Validation of a food frequency questionnaire for assessing dietary nutrients in Brazilian children 5 to 10 years of age. Nutrition.

[7] Hendricks C., Murdaugh C., Pender N. (2006). The Adolescent Lifestyle Profile: Development and psychometric characteristics. J. Natl. Black Nurses Assoc. JNBNA.

[8] Purwaningrum D.N., Sibagariang H.Y.M., Arcot J., Hadi H., Hasnawati R.A., Rahmita R.S., Jayasuriya R. (2018). Validation of a measurement instrument for parental child feeding in a low and middle-income country. Int. J. Behav. Nutr. Phys. Act..

[9] Miller J., Wells L., Nwulu U., Currow D., Johnson M.J., Skipworth R.J.E. (2018). Validated screening tools for the assessment of cachexia, sarcopenia, and malnutrition: A systematic review. Am. J. Clin. Nutr..

[10] Anderson A.S., Bell A., Adamson A., Moynihan P. (2002). A questionnaire assessment of nutrition knowledge—Validity and reliability issues. Public Health Nutr..

[11] Moore J.B., Pawloski L., Baghi H., Whitt K., Rodriguez C., Lumbi L., Bashatah A. (2005). Development and examination of psychometric properties of Self-Care instruments to measure nutrition practices for English and Spanish-speaking adolescents. Self-Care Depend. Care Nurs. Off. J. Int. Orem Soc..

[12] Thiagarajah K., Fly A.D., Hoelscher D.M., Bai Y., Lo K., Leone A., Shertzer J.A. (2008). Validating the Food Behavior Questions from the Elementary School SPAN Questionnaire. J. Nutr. Educ. Behav..

[13] Carlson E.D. (2000). A case study in translation methodology using the Health-Promotion Lifestyle Profile II. Public Health Nurs..

[14] Nunnally J.C., Bernstein I.H. (1994). Psychometric Theory.

[15] Moore J.B., Pawloski L.R., Goldberg P., Kyeung M.O., Stoehr A., Baghi H. (2009). Childhood Obesity Study: A Pilot Study of the Effect of the Nutrition Education Program Color My Pyramid. J. Sch. Nurs..

[16] United States Department of Agriculture Dietary Guidelines|Choose MyPlate. https://www.choosemyplate.gov/dietary-guidelines.

[17] Evers G. Measurement of self-care in clinical practice. Proceedings of the 6th International Self-Care Deficit Nursing Theory Conference.

[18] Stevens J.P. (2009). Applied Multivariate Statistics for the Social Sciences.

[19] Pedhazur E.J., Schmelkin L.P., Student (1991). Measurement, Design, and Analysis: An Integrated Approach.

[20] Van Bokhorst-de van der Schueren M.A.E., Guaitoli P.R., Jansma E.P., de Vet H.C.W. (2014). Nutrition screening tools: Does one size fit all? A systematic review of screening tools for the hospital setting. Clin. Nutr..

[21] Tabachnick B.G., Fidell L.S. (1996). Using Multivariate Statistics.

